# A nonfunctional copy of the salmonid sex-determining gene (*sdY*) is responsible for the “apparent” XY females in Chinook salmon, *Oncorhynchus tshawytscha*

**DOI:** 10.1093/g3journal/jkab451

**Published:** 2022-01-03

**Authors:** Sylvain Bertho, Amaury Herpin, Elodie Jouanno, Ayaka Yano, Julien Bobe, Hugues Parrinello, Laurent Journot, René Guyomard, Thomas Muller, Penny Swanson, Garrett McKinney, Kevin Williamson, Mariah Meek, Manfred Schartl, Yann Guiguen

**Affiliations:** 1 INRAE, LPGP, Rennes 35000, France; 2 Physiological Chemistry, Biocenter, University of Wuerzburg, Wuerzburg 97074, Germany; 3 Institut de Génomique Fonctionnelle, IGF, CNRS, INSERM, Univ. Montpellier, Montpellier 34094, France; 4 GABI, INRAE, AgroParisTech, Université Paris-Saclay, Paris 75005, France; 5 Julius-von-Sachs-Institute, Department of Molecular Plant Physiology and Biophysics, University of Wuerzburg, Wuerzburg 97082, Germany; 6 Environmental and Fisheries Sciences Division, Northwest Fisheries Science Center, National Marine Fisheries Service, National Oceanic and Atmospheric Administration, Seattle, WA 98112, USA; 7 Molecular Genetics Laboratory, Washington Department of Fish & Wildlife, Olympia, WA 98501, USA; 8 Atreca, Inc., San Carlos, CA 94070, USA; 9 Dept. of Integrative Biology, AgBio Research, and Ecology, Evolution, and Behavior Program, Michigan State University, East Lansing, MI 48824, USA; 10 The Xiphophorus Genetic Stock Center, Department of Chemistry and Biochemistry, Texas State University, San Marcos, TX 78666, USA; 11 Department of Developmental Biochemistry, Biocenter, University of Wüerzburg, Wuerzburg 97074, Germany

**Keywords:** *sdY*, sex determination, salmonids, sex reversal, XY females

## Abstract

Many salmonids have a male heterogametic (XX/XY) sex determination system, and they are supposed to have a conserved master sex-determining gene (*sdY*) that interacts at the protein level with Foxl2 leading to the blockage of the synergistic induction of Foxl2 and Nr5a1 of the *cyp19a1a* promoter. However, this hypothesis of a conserved master sex-determining role of *sdY* in salmonids is challenged by a few exceptions, one of them being the presence of naturally occurring “apparent” XY Chinook salmon, *Oncorhynchus tshawytscha*, females. Here, we show that some XY Chinook salmon females have a *sdY* gene (*sdY-N183*), with 1 missense mutation leading to a substitution of a conserved isoleucine to an asparagine (I183N). In contrast, Chinook salmon males have both a nonmutated *sdY-I183* gene and the missense mutation *sdY-N183* gene. The 3-dimensional model of SdY-I183N predicts that the I183N hydrophobic to hydrophilic amino acid change leads to a modification in the SdY β-sandwich structure. Using in vitro cell transfection assays, we found that SdY-I183N, like the wild-type SdY, is preferentially localized in the cytoplasm. However, compared to wild-type SdY, SdY-I183N is more prone to degradation, its nuclear translocation by Foxl2 is reduced, and SdY-I183N is unable to significantly repress the synergistic Foxl2/Nr5a1 induction of the *cyp19a1a* promoter. Altogether, our results suggest that the *sdY-N183* gene of XY Chinook females is nonfunctional and that SdY-I183N is no longer able to promote testicular differentiation by impairing the synthesis of estrogens in the early differentiating gonads of wild Chinook salmon XY females.

## Introduction

Genetic sex determination is a widespread mechanism in vertebrates controlled by master sex-determining genes acting on the top of a genetic cascade, ultimately leading to male and female phenotypes (Bachtrog et al. 2014). Despite recent technological improvements in genome sequencing and genetics, and despite increasing discoveries of master sex-determining genes or candidates in vertebrates, only a handful have been functionally characterized to a certain extent. In fish, most of the currently known sex-determining genes are poorly conserved, for instance *dmrt1bY* that is only found in *Oryzias latipes* and *Oryzias* *curvinotus* (Schartl 2004) or *amhr2Y* in some *Takifugu* species (Ieda et al. 2018). In contrast, most salmonids have been found to harbor the same unusual sex-determining gene named *sdY* (*sexually dimorphic on the* Y) (Bertho et al. 2021). In rainbow trout (*Oncorhynchus mykiss*), this gene, which arose from a duplication of the *irf9* immune-related gene, is necessary and sufficient to drive testicular differentiation (Yano et al. 2012, 2014). SdY triggers its action by interacting with the conserved female differentiation factor Foxl2 (Bertho et al. 2016), ultimately preventing the regulation of estrogen synthesis needed for ovarian differentiation (Bertho et al. 2018). As *sdY* is genotypically tightly sex-linked to male development in most salmonid species, it has been suggested that *sdY* could have been conserved over 50–90 million years as the only sex-determining gene of all extant salmonids (Yano et al. 2013). However, this evolutionary conservation hypothesis has been challenged by some unresolved exceptions to the rule (Yano et al. 2013; Cavileer et al. 2015; Larson et al. 2016; Podlesnykh et al. 2017; Ayllon et al. 2020; Brown et al. 2020), suggesting that *sdY* can be nonfunctional in some salmonids, or that environmental factors override the function of *sdY*. In cases of *sdY*-negative males, the sex-linkage discrepancies could be explained to be the result from neomasculinization of XX females. This phenomenon has been reported in many fish species including some salmonids (Quillet et al. 2002; Valdivia et al. 2013). But some studies also report the existence of *sdY*-positive females. This is more difficult to reconcile with the idea that *sdY* is still acting as a male sex-determining gene in these species. One of the best documented case of such exceptions to the rule is the existence of “apparent” XY females in wild populations of Chinook salmon, *Oncorhynchus tshawytscha*. In this species, discrepancies between genotypic and phenotypic sex have been found with some phenotypic females being described with a male genotype, as deduced from the presence of the male-specific marker, *OtY1* (Williamson and May 2002). These XY females are fully fertile and cannot be distinguished phenotypically from genetically normal XX females (Williamson and May 2002). This observation has been reported several times and in different Northwest Pacific regions including the Columbia river (Nagler et al. 2001; Chowen and Nagler 2004), Alaska (Yano et al. 2013; Cavileer et al. 2015), Idaho, Washington (Cavileer et al. 2015), and California (Williamson and May 2002, 2005; Williamson et al. 2008). The incidence of these XY females varies between 20% and 38% in Central Valley rivers while ranging between 0% and 14% under hatchery conditions (Williamson and May 2002). In addition, independent surveys found proportions of wild-caught XY females ranging from 12% (Cavileer et al. 2015) up to 84% (Nagler et al. 2001). These studies indeed raised many important concerns about the underlying mechanisms of the observed “outliers” and their impact on wild and hatchery Chinook salmon populations. Multiple, independent hypotheses were proposed to explain this genotype/phenotype incongruence, including the possibility that Chinook salmon could be feminized due to endocrine-disruptor chemicals or pollutant exposition (Nagler et al. 2001). Such hypotheses were later excluded using artificial crosses between genotypically normal males (XY) and XY females, showing that half of their phenotypic female offspring were also XY females (Williamson and May 2005) based on Y-chromosome markers (Du et al. 1993; Noakes and Phillips 2003). In addition, fluorescence in situ hybridization revealed that XY-female Chinook salmon in California are not the product of a Y-chromosome to autosome translocation (Williamson et al. 2008) and that these XY females are positive for the *sdY* gene (Cavileer et al. 2015).

We explored sex determination in these “apparent” XY Chinook salmon females to investigate if *sdY* could be still considered as the master sex-determining gene in this species, despite the existence of *sdY*-positive phenotypic females. We amplified and sequenced *sdY* gene between exons 2 and 3 of XY Chinook salmon females and found that they have a missense mutation in the third exon of the *sdY* gene that produces a single amino acid change (I183N) in a highly conserved position of the SdY protein, while males have the wild-type copy of SdY. The mutation modifies the 3-dimensional (3D) structure. The mutant SdY I183N protein is less stable than the wild-type SdY and is affected in its ability to interact with its protein partner, Foxl2 (Bertho et al. 2018). This failure in turn leads to the inability to repress the *cyp19a1a* promoter and thereby to suppress female development. Altogether our results suggest that the *sdY-N183* copy in XY Chinook salmon females is inactive and cannot block the female pathway in the same way as the wild-type *sdY* gene. Our results provide an explanation for the existence of naturally occurring XY Chinook salmon females and support the role of *sdY* as the master male sex-determining gene of Chinook salmon.

## Materials and methods

### Chinook samples genotyping

Family panels and genetic samples were the same as the ones described in Williamson and May (Williamson and May 2005) and were produced from a fall-run Californian Chinook population harvested at the Merced Hatchery. These family panels include crosses from a genotypic/phenotypic female (female XX 84) with a XY male (male B) and a phenotypic female with a XY genotype (Female 126 and Female 118) with a XY male (male D and male C). The OtY1, OtY2, and GH-pY genetic markers were used to assess the sex of each individual (parents and progeny). PCR sequencing analysis of exon 2, intron 2, and exon 3 was performed using a long-range PCR protocol and primers designed upstream and downstream of the *sdY* Chinook gene sequence (GenBank ID = KC756279.2), followed by targeted Sanger resequencing of these PCR fragments with internal primers for exon 2, intron 2, and exon 3. Long-range PCR were carried out in a final volume of 50 μl containing 0.4 μM of each primers (sdYChinook-F2: TTGGCTCCCAGGAAAACATTTCT; sdYChinook-R1: CAGAACAAACAGCATGAAGTAAGCA), 80 ng gDNA, 1× of 10× AccuPrime buffer II (including dNTPs), and 1.5 μl per reaction of AccuPrime HiFi Taq DNA polymerase. Cycling conditions were as follows: 94°C for 1 min, then 35 cycles of (94°C for 30 s + 64°C for 30 s + 68°C for 6 min).

### Chinook testis RNA-seq

Chinook testis was sampled from an adult male from the Umatilla river (OR), and the testis library was prepared using the TruSeq RNA sample preparation kit, according to manufacturer instructions (Illumina, San Diego, CA) as previously described (Pasquier et al. 2016). These testicular transcriptome reads were mapped on a female Chinook genome assembly (Otsh_v1.0, GCA_002872995.1) plus the *sdY* Chinook gene sequence (GenBank ID = KC756279.2) using BWA (Li and Durbin 2009) with stringent mapping parameters (maximum number of mismatches allowed –aln 2). High-quality reads (MAPQ >40) remapping on the *sdY* gene were visualized and analyzed with the IGV software (Robinson et al. 2011).

### Protein structure prediction

The 3D model of SdY-I183N was predicted using the X-ray structure of the dimeric interferon regulatory factor 5 transactivation domain at 2 Å resolution (PDB ID 3DSH) as template (Chen et al. 2008). The 3D views of SdY-N183 were obtained with PyMOL software (Molecular Graphics System, Version 1.7.4; Schrödinger, LLC).

### Cloning

Plasmids and primers used are listed in Supplementary Tables 2 and 3. A forward primer was generated from the coding sequence of the rainbow trout SdY with a point mutation T/A to mimic the SdY-I183N mutation. Next, the amplified fragment containing the mutation was inserted in pCS2^+^-FLAG:SdY. From this plasmid, a PCR-amplified fragment corresponding to SdY-I183N was inserted into pCS2^+^, pCS2^+^-3xHA, pCS2^+^-3xFLAG, and pGEX-4T1 expression vectors. The pCS2^+^-3xHA:emGFP:SdY-I183N plasmid was obtained by inserting a PCR-amplified fragment corresponding to emGFP in-frame into the EcoRI site between 3xHA and the wild-type SdY.

### Cell culture

Human embryonic kidney (HEK 293T) cells were cultured and maintained in Dulbecco's Modified Eagle Medium (DMEM) (PAN Biotech), supplemented with 10% fetal calf serum (FCS) (PAN Biotech) and 1% penicillin–streptomycin (PAN Biotech) at 37°C with 5% CO_2_. HEK 293 transfections were performed by incubating cells with polyethylenimine (PEI) (100 mg/ml PEI diluted 1:100 in 150 mM NaCl) and the respective plasmids (10 µg for 10-cm dishes, 2 µg for 6-well plates) for 6–8 h into fresh medium. Then, the medium was discarded and fresh medium was added.

### Immunofluorescence

HEK 293T cells were seeded on 6-well plates containing coverslips. After transfection of the corresponding plasmids (pCS2^+^-SdY-N183; pCS2^+^-FLAG:SdY-I183N; pCS2^+^-3xFLAG:SdY-I183N; pCS2^+^-HistoneH2B:mCherry) with or without (pCS2^+^-HA-mCherry-Foxl2b2) for 48 h, cells were fixed in 4% fresh paraformaldehyde for 15 min, extensively washed, and permeabilized with 0.1% Triton X-100 in PBS for 10 min. Then, cells were blocked with 1% BSA during 20 min. Primary antibody (Supplementary Table 4) was incubated overnight at 4°C. After extensive washes with PBS, cells were incubated with Alexa 488-conjugated secondary antibodies in 1% BSA for 1 h, followed by Hoechst 33342 (Invitrogen) staining for 5 min (1 μg/ml final concentration). Cells were mounted using Mowiol 4-88 (Roth). Confocal images were acquired under a Nikon Eclipse C1 laser-scanning microscope (Nikon), fitted with a 60× Nikon objective (PL APO, 1.4 NA), and Nikon image software. Images were collected at 1,024 × 1,024 pixel resolution. The stained cells were optically sectioned in the *z*-axis. The step size in the *z*-axis varied from 0.2 to 0.25 mm to obtain 50 slices per imaged file. All experiments were independently repeated several times at least 3 times. Cytoplasmic localization was counted when the main source of signal comes from the cytoplasm. A nucleocytoplasmic localization was counted when a strong signal was detected in both cytoplasm and nucleus. In a same way, a nuclear localization was counted when the signal was detected in the nucleus and when the signal follows the pattern of fluorescence intensity.

### Western blotting

Cells were lysed in a HEPES-based lysis buffer [20 mM HEPES (pH 7.8), 500 mM NaCl, 5 mM MgCl_2_, 5 mM KCl, 0.1% deoxycholate, 0.5% Nonidet-P40, 10 mg/ml aprotinin, 10 mg/ml leupeptin, 200 mM sodium orthovanadate, 1 mM phenylmethanesulphonylfluoride and 100 mM NaF] for 3 h. Cell debris was pelleted by centrifugation for 15 min at 16,000*g*. Cell lysate protein concentration was measured with a Bradford assay (Cary 50 Spectrophotometer, Varian). The protein lysates (30–50 μg) were resolved by SDS-PAGE on 12% Tris-glycine gels followed by transfer to nitrocellulose membranes. Unspecific binding was blocked with 5% BSA in TBST (10 mM Tris, pH 7.9; 150 mM NaCl; 0.1% Tween) for 1 h at room temperature. Incubation with primary antibodies was performed overnight at 4°C. After 3 washes with TBST, HRP-conjugated antibodies were incubated with blocking solution for 1 h. Following the washes, membranes were incubated with SuperSignal West Pico Chemiluminescent Substrate (Thermo Scientific) for 1 min. The signal from the membranes was detected using the Photo Image Station 4000MM (Kodak). At least 2 independent experiments were performed and representative protein blot images are shown. Quantitative analysis was performed with ImageJ 1.48v software (www.imagej.nih.gov).

### Cycloheximide treatment

HEK 293T cells were transfected either with 3xHA-SdY or 3xHA-SdY-I183N with or without the 3xFLAG-tFoxl2b2 expression vector. Forty-eight hours posttransfection, cells were treated with 50 µM of the protein synthesis inhibitor cycloheximide (Calbiochem), or ethanol as vehicle control during 4 or 8 h. Untreated cells (0 h) and treated cells were harvested and subjected to cell lysis followed by SDS-PAGE and Western Blot as described above.

### MG132 treatment

HEK 293T cells were transfected either with 3xHA:SdY or with 3xHA:SdY-I183N with or without the 3xFLAG:Foxl2b2 expression vector. Forty-eight hours posttransfection, cells were treated with 20 µM of a proteasome inhibitor, i.e. MG132 (Merck) or DMSO as vehicle control during 8 h. Untreated cells (0 h) and treated cells were harvested and subjected to cell lysis followed by SDS-PAGE and Western blot as described above.

### Luciferase assay

HEK 293T cells were transfected using PEI with the following plasmids: 0.3 µg of pGL3-*Olacyp19a1a* sequence (kindly provided by D. Wang Deshou); 0.05–0.4 µg of pCS2^+^-SdY-I183N expression plasmid; 0.05–0.4 µg of pCS2^+^-OlaFoxl2; 0.1 µg of pcDNA3.1-OlaNr5a1; and 0,1 µg of pTK-Renilla used for calibration. Each experiment was performed with 1.0 µg final amount. Adjustments were made with empty vector (pCS2+) accordingly. Firefly luciferase and *Renilla* luciferase readings were obtained using the Dual-Luciferase Reporter Assay System (Promega) and LUMAT LB 9501 luminometer (Berthold Technologies GmbH & Co. KG, Bad Wildbad, Germany).

### Statistical analysis

Data were analyzed using a 2-sided unpaired Student’s *t*-test. In addition, luciferase assay was subjected to 1-way ANOVA with post hoc Dunnett tests. Significant differences are symbolized in figures by asterisks if *P* < 0.001 (***), *P* < 0.05 (**), *P* < 0.01 (*), or N.S. if not significant.

## Results

### XY Chinook salmon females bear a missense mutated copy of *sdY*

**Fig. 1.**
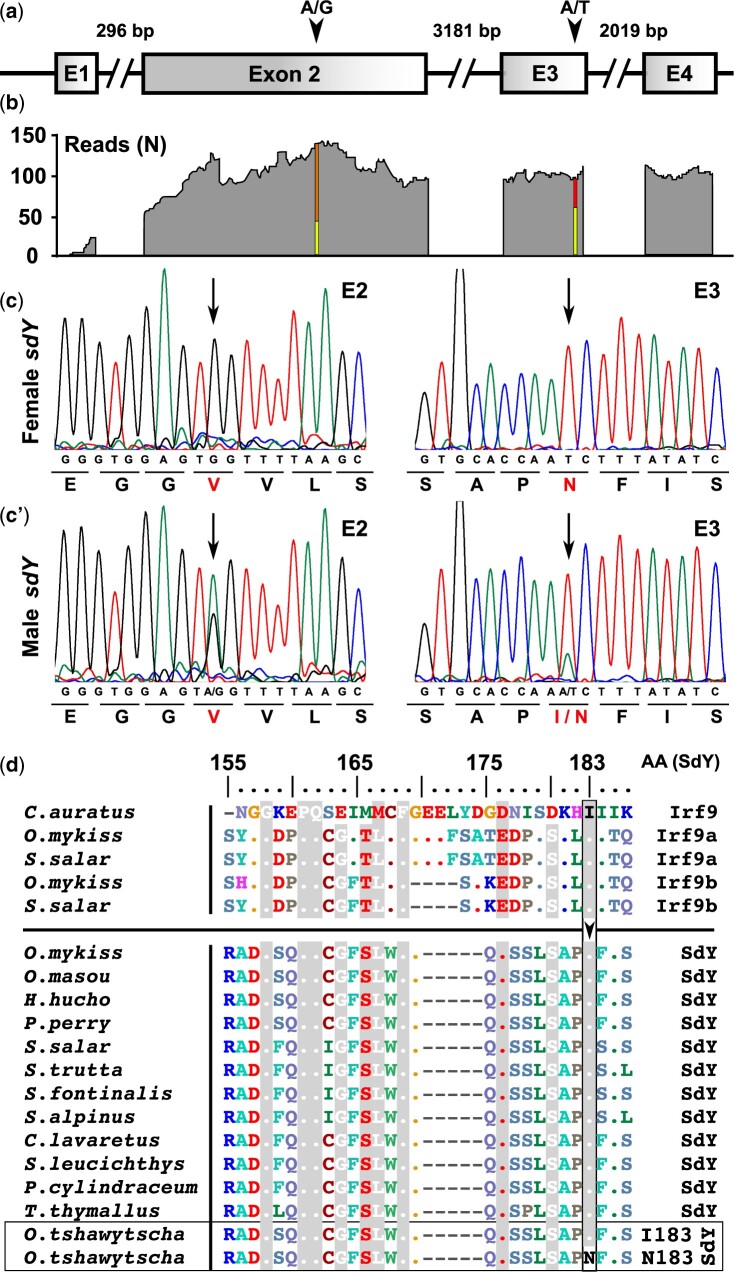
XY Chinook salmon females have a missense mutation in a conserved position of the *sdY* coding sequence. a) Schematic representation of Chinook salmon *SdY* sequence with its 4 exons depicted as square boxes (E1–E4) and the introns as broken lines with intron sizes (bp). b) Remapping of transcriptome reads (*N* = number of raw remapped reads) from a chinook male testis revealed 2 SNVs (A/G and A/T) in the coding region of the *sdY* gene. Representative sequencing chromatograms of parts of the genomic *sdY* coding sequencing containing SNVs in XY females c) and XY males c′) leading to a synonymous mutation in exon 2 (A/G) and a missense mutation in exon 3 (A/T). d) Alignment of Irf9a, Irf9b, and SdY protein sequences in different salmonid species showing the conservation of isoleucine 183 (I) highlighted in gray color and its modification to asparagine (N) only in XY Chinook salmon females (SdY-I183N).

**Fig. 2.**
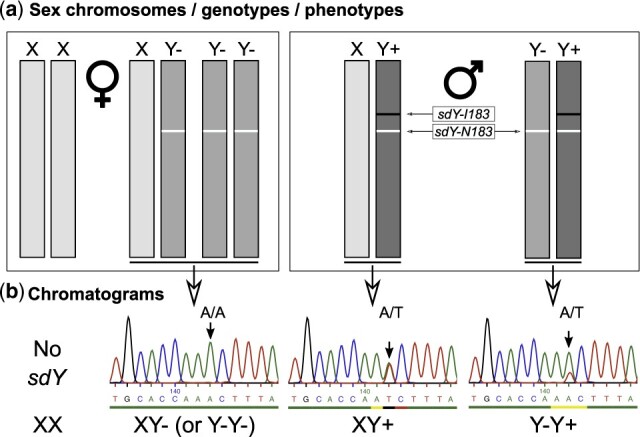
Sex chromosomes, sex genotypes and sex phenotypes in Chinook salmon. a) Schematic representation of sex chromosomes and the hypothetical relation between sex genotypes and sex phenotypes in Chinook salmon. According to our model, phenotypic females can be normal XX females or XY females (XY−) bearing a Y-chromosome (Y−) with a single copy *sdY-N183* gene. Phenotypic males can be XY males (XY+) bearing a Y-chromosome (Y+) with 2 copies of the *sdY* gene, i.e. *sdY-I183* and *sdY-N183*, or Y−Y+ resulting from the crossing of an XY+ male with an XY− female. In turn, a Y−Y+ males crossed with an XY− female can also generate Y−Y− phenotypic females. b) Representative chromatograms of the sequences around the *sdY* I183N mutation (exon 3) in Chinook salmon. XY females (XY−) are homozygotes A/A for the I183N mutation and males are heterozygotes (A/T). Y−Y− females cannot be discriminated from XY− females based on the chromatogram analysis (single A peak of homozygosity in both cases), but XY+ and Y−Y+ could be in theory identified based on the relative peak height of the A/T “pseudo” alleles. With a 1:1 ratio of *sdY-I183* and *sdY-N183*, XY+ males should have an equal A/T peak height and Y−Y+ with a 1:2 ratio of *sdY-I183* and *sdY-N183* should have an A peak height double from the T peak at the same position. Such chromatogram examples are shown in (b) but due to potential variability of the sequencing reactions this genotyping approach was not retained as an accurate approach to discriminate XY− males from Y−Y+ males in our analyses.

The salmonid male sex-determining gene, *sdY*, being only present on the Y-chromosome is generally a single copy gene (Yano et al. 2012, 2013). Whole transcriptome sequencing of a male Chinook salmon testis, however, revealed 2 single-nucleotide variations (SNVs) in the *sdY* coding region (Fig. 1, a and b), suggesting the existence of multiple *sdY* genes or *sdY* alleles. The first SNV is a synonymous A-to-G transition in exon 2 and the second one is an A-to-T transversion in exon 3 that leads to an amino acid change of isoleucine (I) to asparagine (N). To better understand the relation between sex phenotypes and *sdY* genotypes in Chinook salmon, we then checked the presence of *sdY* in XX females, XY females, and XY males using samples from previously described selective crosses between XY or normal XX females with normal XY males (Williamson and May 2005). In agreements with results from wild-caught Chinook salmon (Cavileer et al. 2015), we found that XY females were always *sdY* positive (Supplementary Table 1). Systematic resequencing of XY females revealed that they carried the *sdY* SNVs found in the male testis mRNA in exons 2 and 3 (G/G in exon 2 and T/T in exon 3). In contrast, all males had a double peak at these positions, *i.e.*, A/G in exon 2 and A/T in exon 3, indicating the presence of both *sdY* versions (Fig. 1, c–c′). However, this does not seem to be an indication of allelic variation of a single *sdY* gene as both versions are present in the XY male offspring of a cross of a XY sire with a normal *sdY*-negative XX dam and are not segregating in a Mendelian way in the male offspring (Supplementary Table 1). This suggests the existence of 2 *sdY* genes in XY males that could be tightly linked together on the Y-chromosome (Fig. 2) as almost no recombination was observed in all males (1 homozygote on 81 males) from all the 3 tested progenies. In summary, 2 versions of *sdY* exist in Chinook salmon. The wild-type version would be present only on the Y of males (Y+), while the mutant version would be duplicated on the Y+ of males and present in a single copy on the “apparent” Y (Y−) of XY females (Fig. 2).

The A-to-T substitution in exon 3 leads to a transition from an isoleucine (I183) to an asparagine (N183) at amino acid (AA) position 183 of the SdY sequence. The comparison of all SdY protein sequences and some Irf9 protein sequences (Fig. 1d) available from salmonids show that I183 is highly conserved in both SdY and Irf9, suggesting that it could play an important role in SdY function. These results prompted us to explore if the SdY-I183N mutation could be responsible for the phenotype/genotype discrepancy observed in XY females Chinook salmon.

### The I183N substitution predicts potential local SdY misfolding

**Fig. 3.**
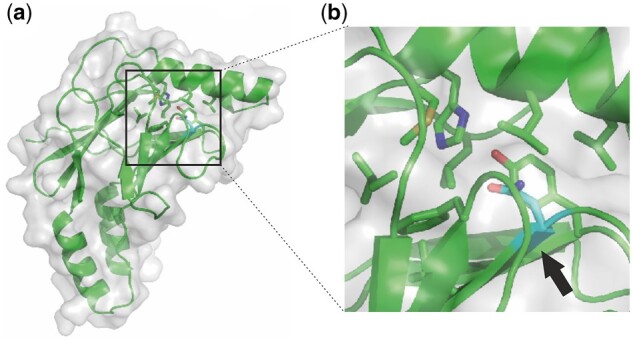
The I183N SdY mutation affects locally the structure of SdY. a) Model of SdY-I183N (green) deduced from the protein-protein interaction domain template of IRF5 (PDB ID 3DSH) embedded in the surface representation (gray). b) Magnification around the asparagine residue (N183) in cyan indicated by a black arrow. The mutation is located at the beginning of the β_7_-strand embedded in a hydrophobic pocket leading to a local misfolding.

To examine more precisely what conformational changes are produced by the I183N substitution, we modelled the SdY-I183N protein 3D structure using the IRF5 domain (PDB code 3dsh) as a template (Fig. 3a). The model revealed that the I183N substitution is localized at the amino terminal end of the β_7_-strand shaping the hydrophobic β-sandwich core element of the protein (Fig. 3b). The mutation induces a hydrophobic (I) to hydrophilic (N) amino acid pattern change likely modifying at least locally the folding of the β-sandwich. We then tried to find the most suitable model of SdY-I183N in which the hydrophilic side chain may have less negative impact on the folding and protein stability. Being exclusively surrounded by hydrophobic amino acids and with a similar size as the isoleucine (I183), it has not been possible to model the asparagine (N183) in an energetically favorable state. The model also suggests that this unfavorable state might impact the local folding of the β-sandwich and the α_1_-helix. Taken together, our protein structure modeling revealed that the I183N substitution affects the local environment of the β-sandwich potentially disturbing SdY folding and leading to a more unstable protein.

### SdY-I183N interaction with Foxl2 is reduced compared to wild-type SdY

**Fig. 4.**
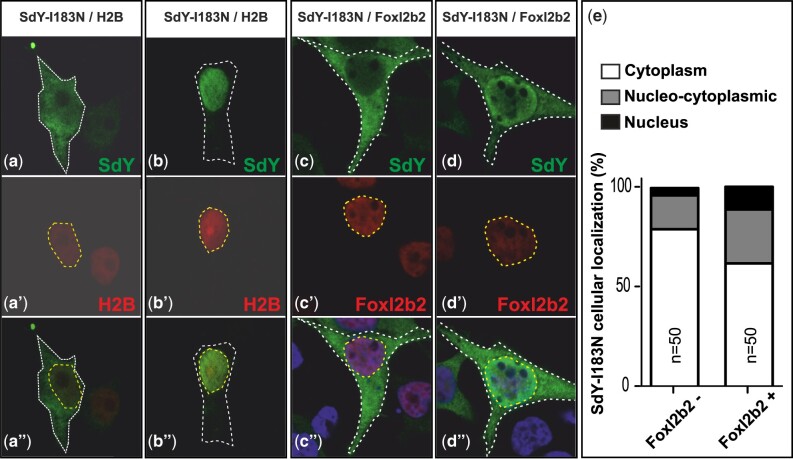
SdY-I183N is localized predominantly in the cytoplasm and is only slightly translocated in the nucleus after cotransfection with Foxl2b2. SdY-I183N alone is mainly detected in the cytoplasm a−a″) with some transfected cells, however, showing a nucleo-cytoplasmic localization [see (e) for quantification of the different localization percentage] and even in some cells a restricted localization in the nucleus b–b″). After cotransfection with Foxl2b2, SdY-I183N remains also mostly cytoplasmic c–c″) with more transfected cells showing a nucleo-cytoplasmic localization [(d−d″) and panel (e) for quantification of the different localization percentage] and a complete localization in the nucleus. e) Quantification of the percentage of transfected cells (measured on 50 transfected cells) with an SdY-I183N localization in the cytoplasm (white bar), in the nucleus (black bar) or with a nucleo-cytoplasmic localization (gray bar) with (Foxl2b2+) or without (Foxl2b2−) cotransfection with Foxl2b2. Human Embryonic Kidney cells (HEK 293T) were transiently cotransfected with rainbow trout SdY-I183N in fusion with 3× Flag tag either with a nucleus marker, i.e. Histone H2B-mCherry (H2B), or a rainbow trout Foxl2b2-mCherry expression construct. Rainbow trout SdY-I183N was detected with an FLAG antibody and the nucleus was stain in red for the H2B construct a′ and b′) or in blue with Hoechst c″ and d″). Scale bar = 5 μm a″–d″).

As Foxl2 has been previously shown to promote SdY translocation from the cytoplasm to the nucleus (Bertho et al. 2018), we further investigated the impact of the I183N substitution on the subcellular localization of SdY in the presence or absence of Foxl2. For this purpose, we engineered the Chinook salmon mutation (I183N) into the rainbow trout SdY protein (SdY-I183N). Like the wild-type SdY, SdY-I183N was predominantly localized in the cytoplasm when transfected alone into human embryonic kidney (HEK 293T) cells (Fig. 4, a–a″ and e). In contrast to the wild-type protein (Bertho et al. 2018), SdY-I183N was also detected in some transfected cells with a nucleo-cytoplasmic localization and even in some cases with a strict nuclear localization (Fig. 4, b–b″ and e). After cotransfection with Foxl2b2, wild-type SdY was mainly localized in the nucleus as previously shown (Bertho et al. 2018). In contrast, SdY-I183N remained predominantly in the cytoplasmic compartment when cotransfected with Foxl2b2 (Fig. 4, b–b″), with a slightly higher percentage of nucleo-cytoplasmic localization (Fig. 3, c–c″) compared to transfections of SdY-I183N alone (Fig. 4e). Altogether, these data show that the cellular localization of SdY-I183N is less restricted than the previously described exclusive cytoplasmic localization of the wild-type protein (Bertho et al. 2018) and that SdY-I183N is also strongly impaired in its ability to be translocated into the nucleus by interaction with Foxl2b2 compared to its wild-type counterpart.

### SdY-I183N is unstable even in the presence of Foxl2b2

**Fig. 5.**
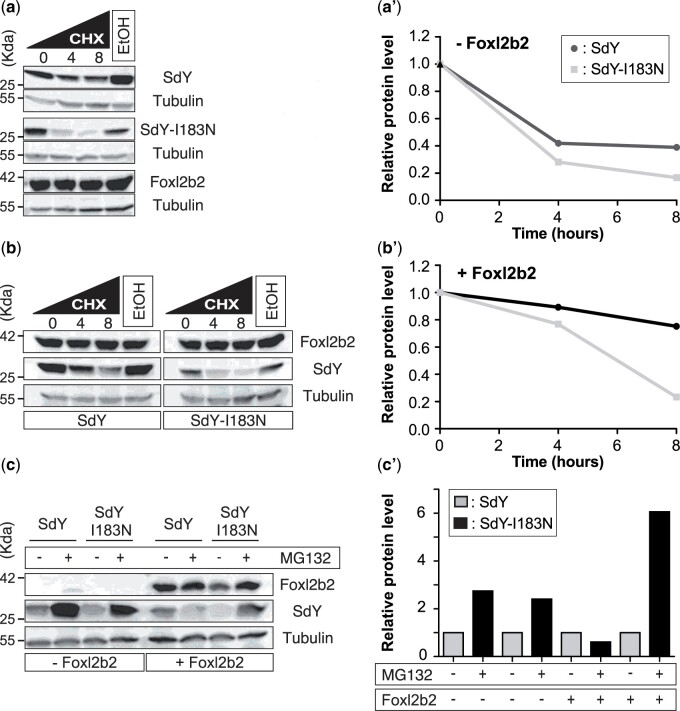
SdY-I183N is unstable even in the presence of Foxl2b2. Cycloheximide (CHX) time course was performed to assess SdY or SdY-I183N stability in the presence or absence of Foxl2b2. HEK cells were transiently transfected with SdY, SdY-I183N, or Foxl2b2 a–a′) alone or with SdY or SdY-I183N in combination with Foxl2b2 b–b′). Cells were treated with 50 μm of CHX and harvest at 4 and 8 h a′ and b′). Lysates were standardized for total protein concentration and expression levels of SdY, SdY-I183N, or Foxl2b2 were detected by Western blotting. Tubulin was blotted as a loading control. Foxl2b2 increased SdY but not SdY-I183N stability b and d). e) Western blot analysis of SdY, SdY-I183N alone, or in combination with Foxl2b2 protein levels following 8 h treatment with proteasome inhibitor MG132. Cells were treated with DMSO [vehicle (control), indicated by a − sign] and MG132 (20 µM, indicated by a + sign). Tubulin was blotted as a loading control f). Quantification of (e).

Because of the local misfolding of SdY-I183N and its lower nuclear translocation following interaction with Foxl2b2, we evaluated the stability of the wild-type and mutant proteins, in the presence or absence of Foxl2b2, by time course treatments with a protein synthesis inhibitor (cycloheximide, Fig. 5, a and b) and a proteasome inhibitor (MG132, Fig. 5c). Both wild-type protein and SdY-I183N expression levels showed a marked decrease 4 h after the beginning of the cycloheximide treatment (Fig. 5, a–a′). Compared to the wild-type protein, SdY-I183N showed reduced expression levels at 4 and 8 h after cycloheximide treatment (Fig. 5, a–a′). After cotransfection with Foxl2b2, expression of both proteins was maintained at relatively high levels 4 h after the beginning of cycloheximide treatment (Fig. 5, b–b′). However, in contrast to the wild-type protein that remained highly expressed at 8 h posttreatment, SdY-I183N expression dramatically decreased (Fig. 5, b–b′). After treatment with the proteasome inhibitor, expression levels of both proteins were roughly doubled (Fig. 5, c–c′). In the presence of Foxl2b2, wild-type SdY protein expression levels were not increased by the proteasome inhibitor treatment. In contrast, SdY-I183N protein expression level was increased 6-fold relative to untreated cells by the treatment (Fig. 5, c–c′). Collectively, this shows that the wild-type SdY protein is stabilized in the presence of Foxl2b2, because most likely the interaction with Foxl2b2 protects it from proteasome-mediated degradation. In contrast, the SdY-I183N protein is much more instable probably because of reduced interaction with Foxl2b2 leading to a higher proteasomal degradation.

### SdY-I183N is unable to repress the *cyp19a1a* promoter

**Fig. 6:**
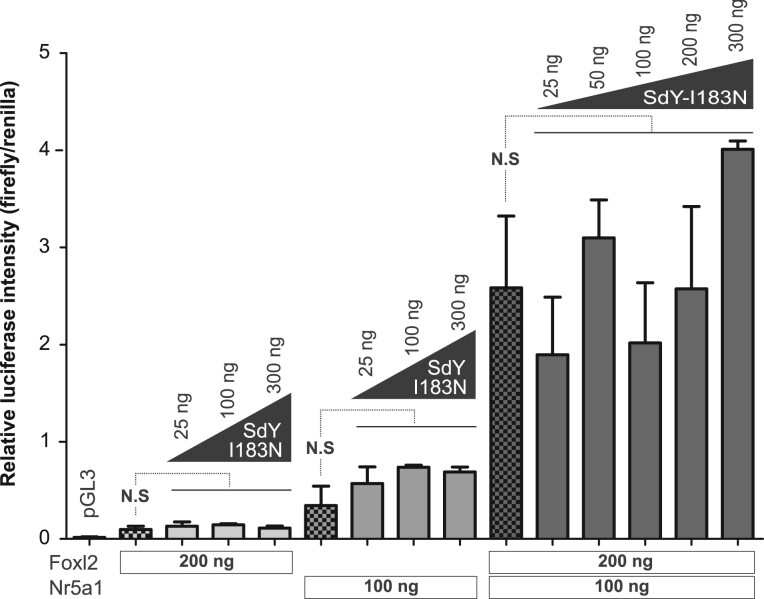
SdY-I183N does not prevent Foxl2/Nr5a1-positive regulation of the *cyp19a1a* promoter (see also Supplementary Fig. 1). The *cyp19a1a* promoter activity (*cyp19a1a* promoter coupled to firefly luciferase) was measured in HEK 293 cells using a luciferase reporter assay and cotransfection of fixed quantities of *nr5a1* (100 ng), *foxl2* (200 ng), and variable quantities (25–300 ng) of *sdY-N183*. Results are calculated from the mean ± SEM of 3 biological replicates in 1 experiment. Statistics were calculated with a one-way ANOVA with post hoc Dunnett tests. N.S: not statistically significant. Empty vector control (pGL3).

To get more insight about how the functionality of SdY-I183N is compromised, we also explored the ability of SdY to repress the *cyp19a1a* promoter in synergy with Foxl2 and Nr5a1 (Bertho et al. 2018). Like wild-type SdY, SdY-I183N is not able to repress the *cyp19a1a* promoter either with Foxl2 alone or Nr5a1 alone (Fig. 6). However, unlike SdY (Supplementary Fig. 1), SdY-I183N is unable to repress the Foxl2 and Nr5a1 synergetic activation of the *cyp19a1a* promoter (Fig. 6), suggesting that *sdY-I183N* is a nonfunctional master sex-determining gene.

## Discussion

Our study identified a missense mutation (I183N) affecting the sex-determining factor SdY in wild XY female Chinook salmon population. The SdY-I183N protein is characterized by a predicted local conformational change in the β-core sandwich, preferential cytoplasmic localization, reduced half-life time, and a lower Foxl2 affinity relative to the wild-type version leading to its inability to repress the *cyp19a1a* promoter.

The isoleucine amino acid at position 183 within the C-terminal domain has a high degree of conservation among the SdY proteins. It is positioned in the protein-protein interaction domain (interferon-associated domain) of its progenitor Irf9 (Yano et al. 2012, 2013). Previous experiments of genetic ablation of *sdY* using zinc fingers nucleases targeting exon 2 resulted in 14 different mutations such as deletion of leucine 43 (L43) that did not lead to sex reversal while the 13 other mutations lead to a clear male-to-female sex reversal (Yano et al. 2014). L43 is present in a linker between 2 β-sheets but not in the β-sandwich. Both amino acids are also conserved in the IAD sequence of Irf9 sequence pointing out a divergence between the conserved primary sequence and the 3D structure. However, this study and our results suggest some crucial amino acid essential for the 3D structure and for the interaction. Such mutations affecting *irf9* have not been described so far.

Taken together, less colocalization with Foxl2 and interaction with lower affinity may be due to the instability of SdY-I183N compared to the wild-type version. Ultimately, the mutated sex-determining factor SdY-I183N was not able to act accurately as a repressor of Foxl2 activity. Consistently with these data, SdY-I183N in XY females would not be effective for inducing testicular differentiation.

Here, we bring a potential explanation for the natural sex reversal observed in some wild Chinook salmon populations. Wild fish sex reversals have also been discovered in other species such as Japanese medaka (*O. latipes*) (Matsuda et al. 2002; Shinomiya et al. 2004; Otake et al. 2006). In medaka, 2 types of mutations affected the sex-determining gene *dmy*/*dmrt1bY* and lead to a XY male-to-female sex reversal. One type of mutations triggered a low expression of *dmrt1bY* insufficient to tilt the balance toward testis development and the second mutation type affected the amino acid sequence leading to a frameshift and an inactivated Dmrt1bY protein (Otake et al. 2006). Naturally occurring sex reversals were also observed in Nile tilapia (*Odontesthes* *niloticus*) in different Kenyan lakes (Baroiller and D'Cotta 2016) and in pejerrey (*Odontesthes* *bonariensis*) from the Lake Kasumigaura in Japan (Yamamoto et al. 2014), but the molecular and/or environmental mechanisms involved in these sex reversals have not been revealed yet.

Interestingly, some incongruences between the genotype and phenotype were also described in wild populations in Sockeye salmon (*Odontesthes* *nerka*), (Larson et al. 2016) and Atlantic salmon (*Salmo* *salar*)

(Eisbrenner et al. 2014; Ayllon et al. 2020; Brown et al. 2020) and in some different species of Pacific salmons (Podlesnykh et al. 2017). In the Salmonidae family, a deeper analysis of *sdY* sequences would be needed to explore if some *sdY* mutations could be responsible for these observed genotype/phenotype mismatches.

Of note, the SdY-I183N mutation has been evaluated in our study at the individual scale within a few families, but its frequency at the population level has not been thoroughly characterized in Chinook salmon. Further information would then be needed to assess the impact of this mutation across populations considering that already some rivers have about 10% of “apparent” XY sex-reversed fish (Williamson et al. 2008; Cavileer et al. 2015). Interestingly, Cavileer et al. (2015) published a Chinook salmon *sdY* genomic DNA sequence (GenBank: KC756279) from Tozitna River, Alaska and a *sdY* cDNA sequence (GenBank: KF006343) from embryonic males from Clearwater River, Idaho. The alignment of those sequences with ours revealed the same nucleotide substitution (A/T) in exon 3 but not the (A/G) substitution in exon 2. We also report in the present study RNA-seq data from a Chinook male from the Umatilla river in Oregon that also has these 2 mutations. The presence of this nonfunctional mutation in 3 different populations across the North America coast (Alaska, Oregon and California) supports the hypothesis that this mutational event occurred before the establishment of these different populations and that this mutation could be widespread in many Chinook populations. However, we cannot exclude that other *sdY* mutations could be present over the whole range of Chinook distribution and the analysis of more populations and individuals over the whole range of Chinook distribution would be needed to get a clear picture of the origin and the percentage of fish impacted by this *sdY-I183* mutation. The inactive *sdY* copy as a nonfunctional gene should accumulate further mutations quite rapidly and should show many features of gene decay. However, the propagation of the mutation requires that sex-reversed females maintain similar reproductive capacity and fitness as wild type (Senior et al. 2012) and/or that the frequency of the Y− chromosome increases by genetic drift.

The origin of Y− chromosome, which has only the defective *sdY* version, remains unsolved and a possible influence of the genomic environment of this mutated *sdY* gene is unknown and could also affect its functionality. However, the salmonid sex determination locus containing the wild-type *sdY* has been assigned features of a “jumping locus” behaving like a giant mobile element (Faber-Hammond et al. 2015). Thus, an additional duplication of the *sdY* locus, followed by the fixation of an inactivating mutation could be a likely origin of the defective *sdY-N183* gene. High-quality whole genome sequencing of a XY chinook salmon female would be now required to better characterize the genomic environment of this mutated *sdY* gene and the implementation of an accurate assay such as PCR or qPCR followed by sequencing to test the presence/absence of the mutation would be also helpful for both aquaculture, stock management, population genetics, and conservation biology of this species (Yano et al. 2013).

In conclusion, we demonstrated that some wild Chinook salmon harbor a copy of the sex-determining gene sdY gene, which, due to a missense mutation, lost the ability for testis determination and explains the genetic status of XY “apparent” male-to-female sex reversals. We show that this mutant Y is not effective anymore and behaves like an X-chromosome. So far except for *sdY*, no gene promoting maleness expressed as early as *sdY* has been identified in the Y-specific region of salmonids. Also, no gene present on the X has been shown to be lost from the male specific on the Y-chromosome (MSY). Despite that true sex reversal has been described for salmonids species (Davidson et al. 2009), Chinook XY females are not bona-fide sex reversals (i.e. female-to-male inversion resulting in XX males). Their genome does not harbor a gene that would induce male development; thus, their phenotype reflects accurately their genotype.

## Data availability

The Chinook testis RNA-seq sequences are available at GenBank Sequence Read Archive under the accession number: SRX4998097. Supplementary Material is available via the GSA figshare portal: https://doi.org/10.25387/g3.15066420.
